# Occipital Alpha Connectivity During Resting-State Electroencephalography in Patients With Ultra-High Risk for Psychosis and Schizophrenia

**DOI:** 10.3389/fpsyt.2019.00553

**Published:** 2019-08-16

**Authors:** Tiantian Liu, Jian Zhang, Xiaonan Dong, Zhucheng Li, Xiaorui Shi, Yizhou Tong, Ruobing Yang, Jinglong Wu, Changming Wang, Tianyi Yan

**Affiliations:** ^1^School of Life Science, Beijing Institute of Technology, Beijing, China; ^2^Intelligent Robotics Institute, School of Mechatronical Engineering, Beijing Institute of Technology, Beijing, China; ^3^College of Computer Science and Communication Engineering, Jiangsu University, Zhenjiang, China; ^4^Beijing Key Laboratory of Mental Disorders, Beijing Anding Hospital, Capital Medical University, Beijing, China

**Keywords:** schizophrenia, ultra-high risk for psychosis, alpha rhythm, functional connectivity, occipital lobe

## Abstract

Schizophrenia patients always show cognitive impairment, which is proved to be related to hypo-connectivity or hyper-connectivity. Further, individuals with an ultra-high risk for psychosis also show abnormal functional connectivity-related cognitive impairment, especially in the alpha rhythm. Thus, the identification of functional networks is essential to our understanding of the disorder. We investigated the resting-state functional connectivity of the alpha rhythm measured by electroencephalography (EEG) to reveal the relation between functional network and clinical symptoms. The participants included 28 patients with first-episode schizophrenia (FES), 28 individuals with ultra-high risk for psychosis (UHR), and 28 healthy controls (HC). After the professional clinical symptoms evaluation, all the participants were instructed to keep eyes closed for 3-min resting-state EEG recording. The 3-min EEG data were segmented into artefact-free epochs (the length was 3 s), and the functional connectivity of the alpha phase was estimated using the phase lag index (PLI), which measures the phase differences of EEG signals. The FES and UHR groups displayed increased resting-state PLI connectivity compared with the HC group [F(2,74) = 10.804, p < 0.001]. Significant increases in the global efficiency, the local efficiency, and the path length were found in the FES and UHR groups compared with those of the HC group. FES and UHR showed an increased degree of connectivity compared with HC. The degree of the left occipital lobe area was higher in the UHR group than in the FES group. The hypothesis of disconnection is confirmed. Furthermore, differences between the UHR and FES group were found, which is valuable for producing clinical significance before the onset of schizophrenia.

## Introduction

Schizophrenia (SZ) is a psychiatric disorder characterized by multiple symptoms, such as positive symptoms, negative symptoms, and cognitive symptoms (1). The neurocognitive deficits, such as verbal memory and vigilance, and social cognitive deficits, such as emotion expression and interpersonal relationships, seriously and continuously affect the normal lives of SZ patients (2–4). Researches on different stages of SZ are helpful to the early diagnosis and treatment of SZ. The stages include first-episode schizophrenia (FES), chronic SZ, and ultra-high risk for psychosis (UHR, also known as clinical high risk), depending on cognitive loss and morbidity (5, 6). Among them, UHR is considered the preclinical stage of SZ. Many studies have focused on UHR, with the aim of early detection and intervention to maximize the patient’s functional performance and to preserve a life of the highest possible quality (7, 8). However, few studies compared the different brain activation patterns in FES with the patterns in UHR (9, 10).

Electroencephalography (EEG) is a non-invasive and low cost way to detect the brain activation patterns in severe mental illness (11, 12). In addition to having a low cost, EEG has a millisecond temporal resolution and the different oscillation frequencies of EEG are related to different brain functions. A review of resting-state studies revealed that SZ patients have shown the increase of absolute delta (0.5–4 Hz) and theta (4–8 Hz) power, and also the decrease of absolute alpha (8–13 Hz) power (13). The inconsistent results are reported on delta and theta band in two studies (14, 15). Compared to delta and theta band, the decrease of alpha band activity in resting state (eyes closed) is the dominant result in SZ researches (13, 16). The alpha activity is negatively correlated to positive symptoms of SZ patients (17, 18). Besides, the decreased alpha activity can be modulated by transcranial alternating current stimulation. Further, the increase of alpha activity is related to clinical improvement of auditory hallucinations (19). The alpha power is also influenced by verbal working memory task in SZ patients (20). Taken together, alpha band activity is a sensitive marker in the progress of SZ and more non-linear analyses are necessary.

Functional connectivity analysis is a popular non-linear analysis in recent years. Studies have shown that cognitive impairment in schizophrenia is related to hypo-connectivity or hyper-connectivity between brain regions, but the associated mechanism of these abnormalities is still controversial (21, 22). A large number of researches have revealed the abnormal functional connectivity in patients with SZ (21, 23). SZ is not the result of focal brain abnormalities but the result of pathological connections between brain regions. This view has been influential in SZ research (24). Stam and Straaten found insufficient neuronal network organization in patients with SZ (25). In addition to the abnormal changes in overall brain connectivity, local anomalies were also observed. Mp et al. (26) demonstrated very localized network changes in the frontal and temporal areas, maintaining global network properties. Functional connectivity studies on the early stage of SZ also reveal the abnormal cerebro-cerebellar functional connectivity in FES and UHR (27) and the abnormal frontal-occipital network in UHR (28). Compared to healthy controls and early illness SZ, UHR showed specific abnormal patterns in the functional connectivity between the superior frontal regions and calcarine cortex (29) and functional connectivity in the cerebello-thalamo-cortical circuitry across different tasks (30). In a word, high-risk individuals showed intermediate abnormal resting-state functional connectivity patterns measured by coherence between healthy controls and SZ, but the differences were not significant (31). Thus, the construction of functional connectivity and deep analysis of brain network topology need to be strengthened.

Phase synchronization is an effective method to construct the functional connectivity of EEG detection. EEG is ideal for building large functional connectivity networks and for the analysis of various frequencies, especially since it has good time resolution. However, there are certain drawbacks to using a phase synchronization to build a network (28). Volume conduction affects the construction of functional connectivity due to the distance between electrical potential and source generator. A strong false connection is generated because of the positional deviation between the effect of the recording signal (32). An alternative method is generated to measure functional connectivity to solve the problem of false connections using the phase lag index (PLI) (33). PLI has become an effective research indicator for several mental disorders (34). Studies of SZ using PLI to measure functional connectivity based on EEG at rest also have important applications in the field of disease research and engineering (35). SZ patients have obvious reduced functional connectivity strength measured by PLI in alpha band compared to healthy controls (19, 36). FES patients show the decrease of PLI in the low alpha band (8–10 Hz) in the resting state compared to healthy controls (37). Combined PLI with minimum spanning tree, researchers found the decentralized topology characterized by degree centrality in FES (38). Thus, PLI may be effective to construct the functional connectivity and reveal the different patterns in FES and UHR.

It is highly likely that the topological configuration of functional brain networks can be used to evaluate treatment effects, including those related to cognitive function, and, in some cases, can predict the risk of psychosis (16, 39). To construct functional brain network, the use of resting-state EEG avoids the experimental errors caused by participants’ incompatibility and reduces the difficulty of detection (13). This article aims to study the abnormality of brain connections measured by EEG in the early stage of SZ to produce clinical significance before the onset of SZ. In this study, we hypothesize that FES and UHR show abnormal global functional connectivity patterns measured by PLI compared to healthy controls. Furthermore, the abnormal local functional connectivity will be revealed by degree centrality analysis.

## Materials and Methods

### Participants and Data Acquisition

Sixty-nine participants, including 20 patients with FES, 21 UHR, and 28 healthy controls, were recruited in the present study. UHR participants were recruited according to the Criteria of Prodromal Symptoms from the Structured Interview for Prodromal Syndromes (SIPS) (35). FES patients were diagnosed based on the DSM-IV (Diagnostic and Statistical Manual of Mental Disorders, Fourth Edition). Medical professions evaluated the psychiatric symptoms on the Positive and Negative Syndrome Scale (PANSS). The SIPS scale was used to assess the prodromal syndromes of SZ for participants without SZ. The Global Assessment of Functioning (GAF) and MATRICS Consensus Cognitive Battery (MCCB) were assessed in all subjects to evaluate functioning and cognition. In addition, the Calgary Depression Scale for Schizophrenia (CDSS) was used to rate participants’ depressive symptoms. Demographic and clinical details are summarized in **Table 1**. This study was approved by the Ethics Committee of the Beijing Anding Hospital in accordance with the Declaration of Helsinki, and all participants were given informed written consent before the experiment.

**Table 1:** Demographic data.

	HC	UHR	FES	*P* value
N (sex ratio M/F)	28 (19/9)	21 (13/8)	28 (14/14)	p = 0.384^a^
Age (years)	24.14 ± 3.71	24.10 ± 6.56	25.86 ± 7.33	p = 0.876^b^
Education (years)	13.43 ± 3.72	13.76 ± 3.10	12.25 ± 3.30	p = 0.253^b^
IQ	111.91 ± 14.44	107.54 ± 11.41	96.32 ± 13.33	p < 0.001^b^
CDSST	0.14 ± 0.53	1.90 ± 2.41	1.21 ± 1.19	p < 0.001^b^
GAF	86.86 ± 8.99	57.14 ± 13.04	54.75 ± 11.84	p < 0.001^b^
MCCB	45.14 ± 5.91	39.24 ± 5.91	35.20 ± 5.95	p < 0.001^b^
PANSS				
Positive			23.21 ± 6.09	
Negative			20.75 ± 7.43	
General			41.54 ± 6.25	
Total			84.46 ± 13.02	
SIPS				
Positive	0.50 ± 1.53	9.24 ± 3.27		p = 0.001^c^
Negative	0.36 ± 1.10	9.52 ± 5.41		p < 0.001^c^
Disorganization	0.18 ± 0.67	4.57 ± 2.93		p < 0.001^c^
General	0.25 ± 0.65	5.62 ± 3.92		p < 0.001^c^
Total	1.29 ± 3.51	28.95 ± 9.68		p < 0.001^c^

FES, first-episode schizophrenia; UHR, ultra-high risk for psychosis; HC, healthy controls; GAF, global assessment of functioning; IQ, intelligence quotient; MCCB, MATRICS Consensus Cognitive Battery; PANSS, positive, negative, and general psychopathology scale scores; SIPS, Structured Interview for Prodromal Syndromes.

a χ^2^ test.

b One-way ANOVA. P < 0.05 was considered significant.

c Independent samples test.

### EEG Acquisition and Processing

Participants were instructed to sit comfortably, stay awake, and keep eyes closed and calm in a quiet room during the 3-min EEG recording. One hundred twenty-eight electrodes were arranged, and the reference electrode was Cz during the recording. The arrangement of 128 electrodes was the same as in a previous study (40). The sampling rate was 1,000 Hz, and the electrode impedances were less than 5 kΩ during the recording. In order to further improve the signal noise ratio, a 0.1–100 Hz online bandpass filter and a 0.1–45 Hz offline bandpass filter were combined during the recording. Our data were pre-processed with MATLAB R2017a (Mathworks Inc., Natick, MA, United States) with the open source toolbox EEGLAB (Swartz Center for Computational Neuroscience, La Jolla, CA, United States). An independent component analysis was used to remove artefacts (e.g., eye artefacts, muscle artefacts, and electrocardiographic activity) from the data within all channels. For the selection of epoch length, previous studies have shown that 3–16 cycles (for alpha band, about 2 s) are sufficient for PLI analysis (41, 42). In each epoch, the first and last 75 ms need to be removed to avoid distortions caused by bandpass filtering (43). Under the above considerations and for convenience of calculations, pre-processed data were divided into 3-s epochs, totalling 50 epochs, to keep the data consistent. For each epoch, alpha (8–13 Hz) was isolated by bandpass filtering.

### Network Construction

The network synchronization of alpha oscillations was investigated. For each subject, instantaneous phase measures were calculated for each epoch, source, and frequency band by the Hilbert transform. Phase locking was calculated for each EEG sensor pair and frequency with the PLI (33, 44).

$$\text{PLI}=\left|\langle sign(\Delta \phi (t))\rangle \right|=\left|\frac{1}{M}\sum_{k=1}^{M}sign(\Delta \phi (t))\right|$$

Δϕ describes the phase difference of two time series (3-s epochs) recorded from two electrodes and M is the number of epochs, and 50 epochs are obtained for each participant. Since 128 electrodes are applied in the present study, a 128-by-128 adjacency matrix is obtained for each participant, and the mean of the matrix is calculated for the following comparison.

### Theoretical Analysis of the Network Topologies

A 128-by-128 functional network was constructed for each participant by PLI. To further evaluate the global and local topological properties of the network, network parameters, including clustering coefficients, path length, small world, global efficiency, local efficiency, and degree, were calculated by GRETNA (45). Random effects are removed by generating a total of 100 random networks and comparing with the PLI network. In addition, the brain network parameters were calculated with sparsity ranging from 0.05 to 0.5 (the interval is 0.05). The area under the curve was regarded as the normalized brain network parameters to avoid the influence of sparsity threshold and to check for relative network organization. In addition, the degree analysis of the PLI network was performed as an extended EEG analysis to focus on the abnormal brain changes. Higher degree of one node indicated the more interactions in the network for this node or electrode in the present study. Thus, the degree may be a simple but effective measure to detect the abnormality of networks.

### Statistical Analysis

Statistical analyses were performed on SPSS version 25.0 (SPSS, Inc., Chicago, IL, United States). Two-sample t tests and chi-squared tests were performed on the clinical and demographic data to test the significant differences (p < 0.05). Differences among three groups were analyzed *via* one-way ANOVA and *post hoc* unpaired t-tests (Bonferroni corrected). Relationship between the degree of the alpha band and clinical scales was evaluated by Pearson’s correlations (p < 0.05) and Bonferroni correction due to the multiple tests. The region of interest (ROI) was defined by an appropriate ANOVA to evaluate the potential electrodes by group differences on degree centrality. The part of the variance test p < 0.001 was selected as the ROI, and the ROIs were divided into three areas according to the location.

## Results

### Demographic Characteristics

**Table 1** displays the relevant demographic and clinical information. There was no significant group effect on gender [χ^2^ (2,74) = 1.912, p = 0.384], age [F(2,74) = 0.133, p = 0.876], or education [F(2,74) = 1.399, p = 0.253] among all three groups. The intelligence quotient (IQ) significantly differed among the three groups [F(2,74) = 8.999, p < 0.001]. The IQ of healthy controls and UHR participants showed no significant differences. The results of *post hoc* testing showed that FES participants had significantly lower IQ scores than healthy controls (p < 0.001, Bonferroni) or UHR participants (p = 0.032, Bonferroni). The tests that evaluate functioning and cognition, such as GAF [F(2,74) = 68.278, p < 0.001] and MCCB [F(2,74) = 18.881, p < 0.001], showed significant differences among the three groups. The results of *post hoc* testing showed that FES participants had significantly lower GAF than healthy controls (p < 0.001, Bonferroni) or UHR patients (p < 0.001, Bonferroni), while no differences were found between UHR and FES (p = 1.000, Bonferroni). In addition, the test that rates participants’ depressive symptoms, CDSST, showed a significant difference among the groups [F(2,74) = 8.918, p < 0.001]. Several significant differences were found in the CDSST scores of the three groups (UHR vs. HC: p < 0.001; HC vs. FES: p = 0.25, Bonferroni). However, the difference between UHR and FES was not significant (p = 0.331, Bonferroni).

### Global Network Analysis

**Figure 1:**
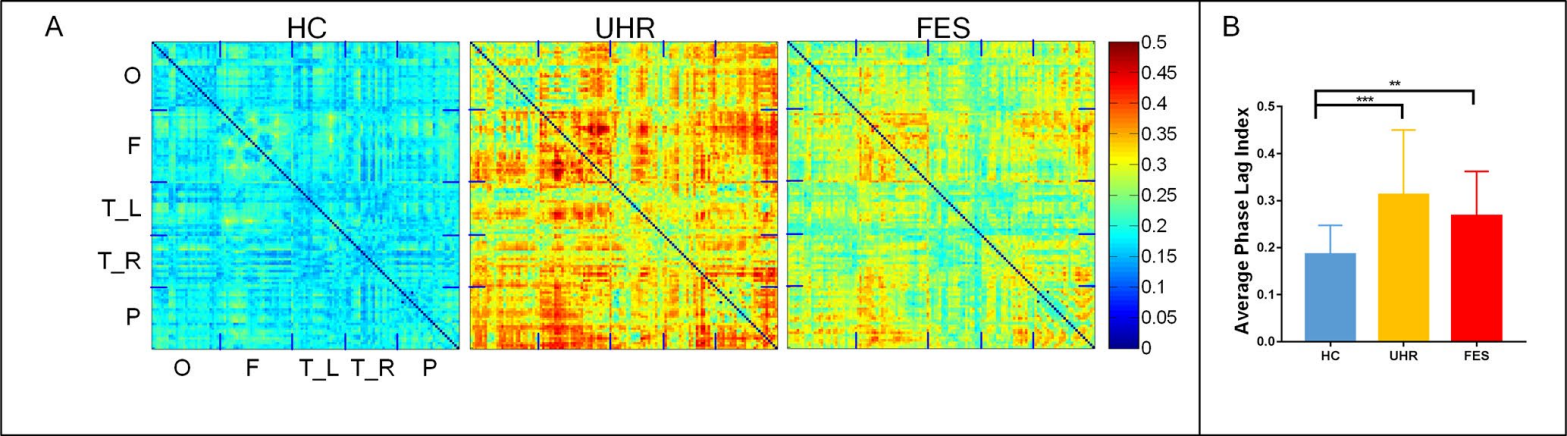
First-episode schizophrenia (FES) and individuals with ultra-high risk for psychosis (UHR) showed an increased spontaneous eyes-closed alpha-band PLI relative to healthy controls (HC). No significant differences were observed for comparisons between the FES and UHR groups. **(A)** Differences can be seen in the PLI distribution of the three groups. The horizontal and vertical axes were electrodes, and the sequences of the electrodes were occipital network (O), frontal network (F), left (T_L) and right (T_R) temporal network, and parietal network (P). **(B)** Global PLI (average of 128 × 128 network) ANOVA results. *Post hoc* t-tests were corrected by Bonferroni. Significantly different results are indicated by asterisks (**: p < 0.01, ***: p < 0.001).

The topographic analyses of the spatial distribution of the alpha frequency in all epochs averaged across the three groups are presented in **Figure 1A**.

The mean PLI was 0.312 ± 0.128 for the UHR group, 0.267 ± 0.09 for the FES group, and 0.183 ± 0.061 for the healthy controls. The group effect on PLI was significant across the three groups [F(2,74) = 10.804, p < 0.001]. The PLI network connection for healthy controls was significantly lower than that for the UHR and FES groups. Differences can be seen in the PLI distribution of UHR and FES, but the differences were not significant. PLI embodies the consistency of the brain network in the phase distribution. In UHR, the brain network presents a diffuse connection distribution. This connection anomaly is concentrated in some areas in FES. In HC, the brain network connections are regionalized and ordered connections.

Brain network parameters were analyzed to assess the group differences in brain network connectivity. Parameters were measured by the area under the curve below different sparsity threshold (from 0.05 to 0.5 with the interval of 0.05), such as clustering coefficient (Cp), path length (Lp), small-worldness (Sigma), global efficiency (Eg), local efficiency (Eloc), and degree centrality. All the group differences are shown in **Table 2**. The discriminative parameters among the groups are emphasized in bold fonts. There are significant interactions for the Eg [p < 0.001, F(2,64) = 6.367], Eloc [p < 0.001, F(2,44) = 3.739], and Lp [p < 0.001, F(2,64) = 4.820] values.

**Table 2:** Alpha network analysis.

	HC	UHR	FES	P value	Post hoc
Assortativity	−0.065 ± 0.058	−0.097 ± 0.059	−0.082 ± 0.061	0.186	
Hierarchy	0.01 ± 0.045	0.009 ± 0.061	0.003 ± 0.053	0.861	
Synchronization	0.013 ± 0.016	0.006 ± 0.008	0.01 ± 0.012	0.130	
**Eg**	0.084 ± 0.039	0.142 ± 0.037	0.13 ± 0.029	** < 0.001**	FES, UHR > HC
**Eloc**	0.088 ± 0.04	0.152 ± 0.044	0.14 ± 0.036	** < 0.001**	FES, UHR > HC
Cp	0.097 ± 0.018	0.105 ± 0.026	0.099 ± 0.022	0.439	
**Lp**	3.229 ± 1.618	1.573 ± 0.335	1.705 ± 0.361	** < 0.001**	FES, UHR < HC
Sigma	0.376 ± 0.044	0.352 ± 0.042	0.368 ± 0.043	0.163	

One-way ANOVA. P < 0.05 was considered significant.

The bolded text indicated the significant difference among groups.

### Degree Centrality Analysis

**Figure 2:**
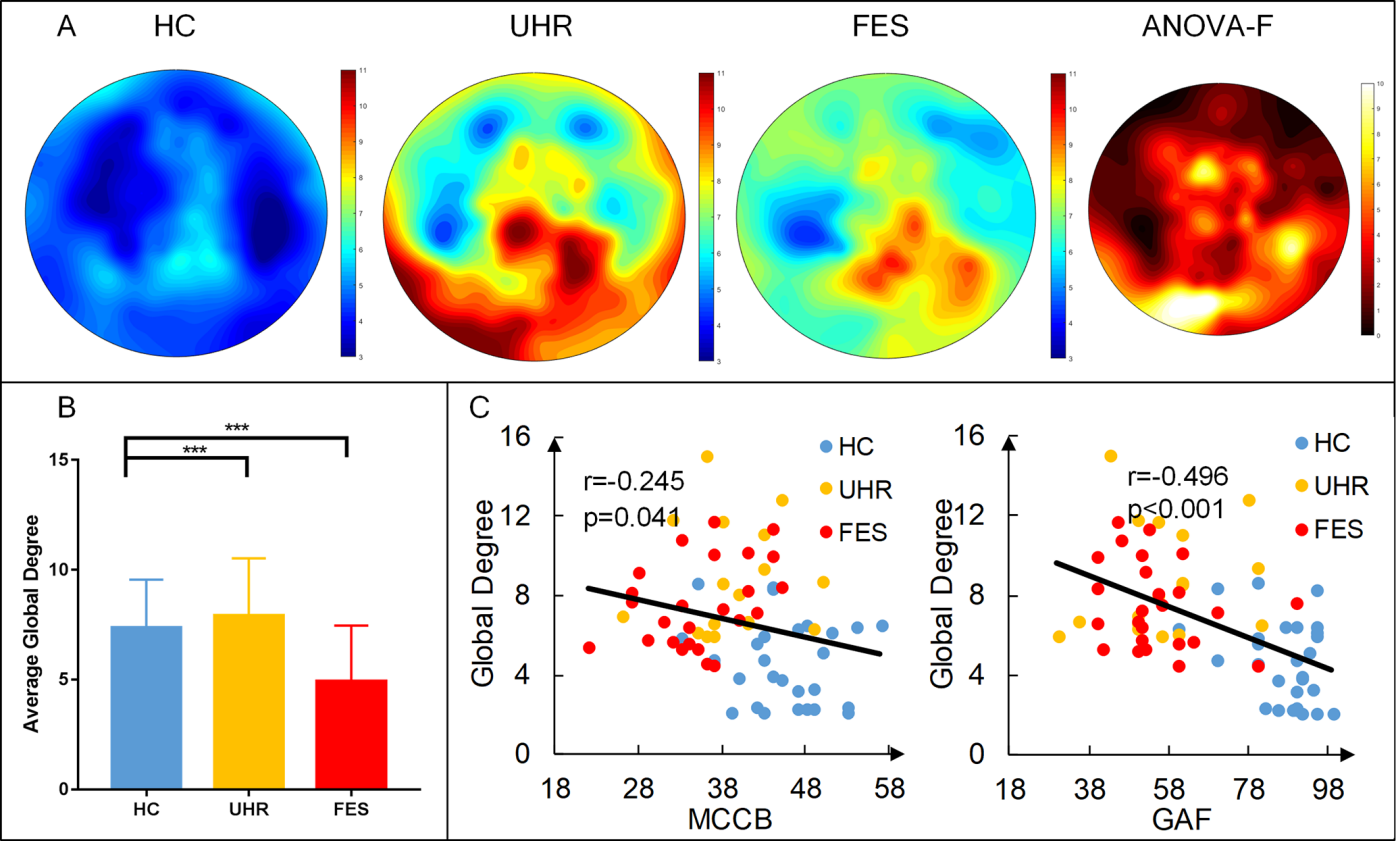
First-episode schizophrenia (FES) and individuals with ultra-high risk for psychosis (UHR) showed an increased degree of connectivity (area under the curve with the sparsity ranging from 0.05 to 0.5 with the interval of 0.05) compared to healthy controls (HC). **(A)** Topography of the average degree of connectivity for the three groups. Rightmost map shows ANOVA results of the three groups. **(B)** Average global degree (average of 128 sites) ANOVA results. *Post hoc* t-tests were corrected by Bonferroni. Significantly different results are indicated by asterisks (***: p < 0.001). **(C)** Correlation of the average degree and cognitive scales. The result shows a negative correlation, which means that the higher the degree, the more damaged the cognitive situation.

The distribution of degrees of centrality (area under the curve with the sparsity ranging from 0.05 to 0.5 with the interval of 0.05) is shown in **Figure 2**. The mean degree centrality was 8.18 ± 2.81 for the UHR group, 7.34 ± 2.16 for the FES group, and 4.64 ± 2.06 for the healthy controls. The group effect on degree centrality was significant across the three groups [F(2,74) = 16.331, p < 0.001]. The degree centrality for healthy controls was significantly lower than that for the UHR and FES groups. Differences could be seen in the degree distribution of UHR and FES, but the differences were not significant. UHR had a higher degree of connectivity in the subtemporal-occipital lobe. In addition, we examined the relation between the average global degrees and cognitive scales in all three groups. The analysis of behaviors showed significant results (MCCB: r = −0.245, p = 0.041; GAF: r = −0.496, p < 0.001). The degree centrality was negatively correlated with the cognitive scale (see **Figure 2C**). We also calculated the correlation within one group (see **Supplementary Table 1**) and between two groups (see **Supplementary Figure 1**).

**Figure 3:**
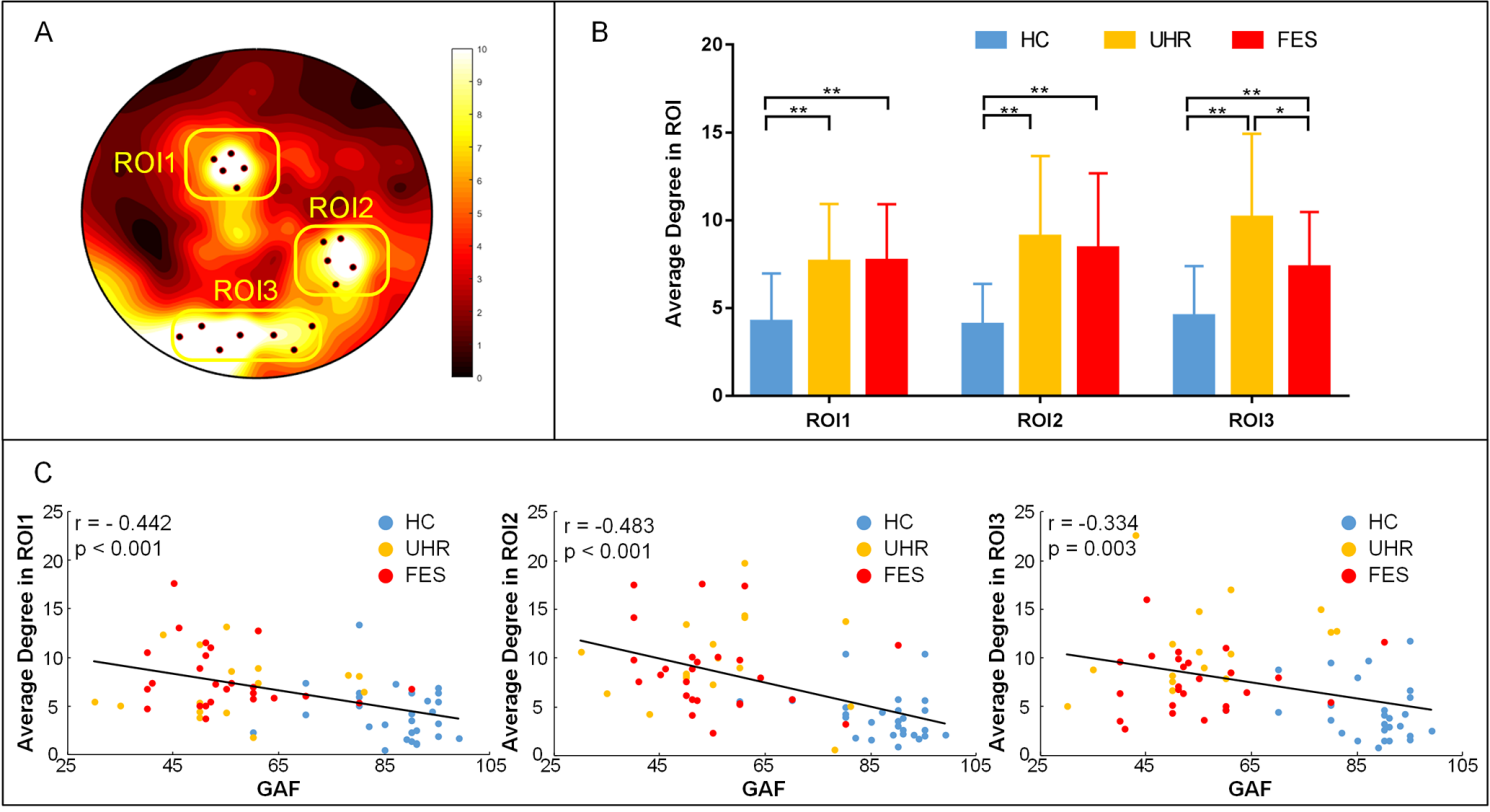
ROI analysis results. **(A)** ROI (black dots) defined as the topography of ANOVA result. Sites whose p < 0.001 were considered. ROI1: superior parietal (electrode number: 12, 13, 19, 20, 24), ROI2: right temporal lobe areas (electrode number: 96, 97, 98, 101, 102), ROI3: occipital lobe areas for three groups (electrode number: 68, 69, 73, 74, 82, 88, 89). The arrangement of 128 electrodes was the same as in a previous study (40). **(B)** Average global degree (average of 128 sites) ANOVA results. *Post hoc* t-tests were corrected by Bonferroni. Significantly different results are indicated by asterisks (*: p < 0.05, **: p < 0.01, ***: p < 0.001). **(C)** Correlation of the average degree and cognitive scales. The results show a negative correlation, which means that the higher the degree, the more damaged the cognitive situation.

To better measure the difference in the degree centrality among the groups, a region of interest (ROI) analysis was applied to the differences among the whole brains of the three groups. The part of the variance test with significance (p < 0.001) was selected as the ROIs, and the ROIs were divided into three groups according to the location (**Figure 3**). The group effect on the degree centrality was significant across the three groups in ROI1 [F(2,74) = 11.154, p < 0.001], ROI2 [F(2,74) = 13.662, p < 0.001], and ROI3 [F(2,74) = 15.004, p < 0.001]. The degree centrality for healthy controls was significantly lower than that for the UHR and FES groups in all three ROIs. No significant differences were found between the UHR and FES groups in terms of ROI1 and ROI2, while the degree centrality of the FES group in ROI3 was significantly lower than that of the UHR group (p = 0.014, Bonferroni).

We examined the relation between the average degree of ROI, where we found statistically significant group differences and cognitive scales in all three groups. In ROI1, the linear regression analysis showed a statistically significant relation between the degrees and the cognitive scales (see **Figure 3C**). We found a statistically significant negative relation between the degree means and the cognitive scales (r = −0.442, p < 0.001). Negative relation between the cognitive scales and the degrees is also found in ROI2 (r = −0.483 p < 0.001) and ROI3 (r = −0.334, p = 0.003). We also calculated the correlation within one group (see **Supplementary Table 1**) and between two groups (see **Supplementary Figures 2** and **3**).

## Discussion

The present study described the abnormal brain disconnection in the FES and UHR groups compared with that in healthy controls. Furthermore, the association with clinical scales has been revealed to demonstrate the relationship between the functional connection results based on EEG and clinical manifestations. Our results showed the increase of functional connectivity mainly in superior parietal, right temporal, and left occipital brain regions. Furthermore, patients showed increased average degree and the degree was related to clinical scales. Differences between the FES and UHR groups have been found on the average degree in ROI3, which may be a potential biomarker of SZ.

Some scholars believe that alpha is related to non-task brain network activity (46), but some studies have shown that alpha-band brain activity is related to cognitive and memory representation, which reflects the performance of attention and semantic memory (47). Based upon these opinions, we believe that task-independent prohibition will help to allocate resources to the task-related areas necessary for optimal task execution. Therefore, we believe that the brain activity of the alpha band in the resting state can reflect the health of the brain to a certain extent. Moreover, several resting-state studies reported abnormalities in the alpha band in SZ (48–51). One of those studies reported a significant correlation between a measure of global network efficiency and cognitive ability in SZ (50).

Two different hypotheses about how abnormal connectivity affects patients are discussed in the field of SZ. For hyperconnectivity hypothesis, it assumed that synapses may fail to be eliminated in development. In contrast, that too many synapses are eliminated is what is believed by hyperconnectivity hypothesis. In this study, the network connection based on PLI was found to be significantly higher in the FES and UHR groups than in healthy controls, and excessive connectivity occurred in these patients. In previous studies, connection enhancements in schizophrenic patients have also been reported (52). The enhancement of local connectivity in the brain of the patient group may be due to impaired connections, which is a notion that was validated in previous studies. Synaptic plasticity may affect the process of connectivity construction. Functional connectivity between neurons may hardly survive from development due to the strength abnormality (53).

The global efficiency and local efficiency in the UHR and FES groups were significantly higher than those in the healthy controls. In addition, path length, which measures brain network connectivity integration, was decreased significantly in the patient group. This result is also reflected in previous studies. Lp for alpha activity was significantly higher in the FES group at rest (25). In summary, there is a problem with the functional integration of schizophrenic patients in brain network connections, which is consistent with previous studies. Further, our results indicate that the UHR group also has the problem of functional connectivity, which may be a more serious problem than that of the FES group.

To further analyze the connection abnormality of schizophrenia, the degree of centrality and ROI analysis were implemented. As mentioned in the above results, the degree of centrality was significantly higher in the FES and UHR groups than in healthy subjects, especially in the superior parietal, right temporal, and left occipital lobe areas. Previous studies have found the structural and functional abnormality of visual cortex in SZ patients by MRI, and the abnormality was related to clinical symptoms (54, 55). As for the abnormality in auditory cortex (56), the abnormality is mainly in the left temporal lobe (57, 58). Similar abnormality of the left temporal area in SZ is also found by PET (59). In the analysis of the degree centrality, the degree of the UHR group was significantly higher than that of the FES group in the occipital lobe areas. Previous studies have shown the abnormality of visual cortical processing in patients with SZ and UHR participants (60). SZ patients also showed other cortical processing dysfunctions (61). In the early stage of SZ, such as UHR participants, cognitive deficits are found (62).

We observed a statistically significant relation between the degree of network and cognitive scores in the three groups (see **Figures 2C**, **3C**). The degree of centrality in the ROI was negatively correlated with the score on the cognitive scale, indicating that the better the cognitive performance of the subject, the lower the degree of centrality. The relevant results in the present study suggest that the cognitive decline in schizophrenic patients is related to their network connectivity. This pattern of results suggests that as the abnormal nodes of the brain network increase, the cognitive ability of patients decreases. The direction of this relation indicates that some individuals with SZ might show a protracted developmental course of network topology.

Our results demonstrated that brain networks estimated at rest can also predict the stratified level of consciousness in patients and predict patient clinical outcomes. However, a current limitation of the EEG-based assessment proposed here stems from the expert intervention required for artefact removal, specifically for inspecting and identifying noisy data and independent components. There have been many recent methodological advances in automating this step (63–65), and in the future, we need more participants and follow-up study to validate our methods and hypothesis.

The current study has identified brain function network defects in patients with FES and clinically high-risk patients. The abnormal global and local functional networks are revealed in the different stages of SZ. The present methods on network construction and analysis, and the results of correlation between the central degree topological measurement and the clinical scales may be helpful in understanding the dysfunction syndrome of SZ.

## Ethics Statement

This study was carried out in accordance with the recommendations of the Ethics Committee of the Beijing Anding Hospital with written informed consent from all participants. All subjects gave written informed consent in accordance with the Declaration of Helsinki. The protocol was approved by the Ethics Committee of the Beijing Anding Hospital.

## Author Contributions

TL participated in experiments, assisted in data analysis, and wrote the paper. JZ analyzed the data and revised the paper. XD analyzed and interpreted the data and wrote the paper. ZL, XS, YT and RY helped with revised the paper and assisted in data analysis. JW provided a schematic of the principle. CW carried out scale evaluation and acquired the data. TY analyzed organized results and approved the final version.

## Funding

This work was supported by the National Key Research and Development Program of China under grant 2017YFB1002504, the National Natural Science Foundation of China (Grant No. 81671776, 61727807, 61633018), the Beijing Municipal Science & Technology Commission (Z181100003118007,，Z191100010618004), research plan for innovation in clinical technology by Beijing Hospitals Authority (XMLX201805),，the Beijing Nova Program (Grant No. Z171100001117057).

## Conflict of Interest Statement

The authors declare that the research was conducted in the absence of any commercial or financial relationships that could be construed as a potential conflict of interest.
