# Supplementary material for: Occipital Alpha Connectivity During Resting-State Electroencephalography in Patients With Ultra-High Risk for Psychosis and Schizophrenia
Source: Front Psychiatry. 2019 Aug 16;10:553. doi: 10.3389/fpsyt.2019.00553 (PMC6706463; doi:10.3389/fpsyt.2019.00553)
Supplement: Supplementary file 1 [file DataSheet_1.docx]

Supplementary Material

Occipital Alpha Connectivity During Resting-State EEG in Patients with Ultra-high Risk for Psychosis and Schizophrenia

Tiantian Liu^1#^, Jian Zhang^2#^, Xiaonan Dong^1^, Jinglong Wu^2^, Changming Wang^3*^, Tianyi Yan^1^*

^1^ School of Life Science, Beijing Institute of Technology, Beijing, China

^2^ Intelligent Robotics Institute, School of Mechatronical Engineering, Beijing Institute of Technology, Beijing, China

^3^ Beijing Key Laboratory of Mental Disorders, Beijing Anding Hospital, Capital Medical University, Beijing, China

**^#^**These authors have contributed equally to this work

*** Correspondence:**Prof. Tianyi Yan
yantianyi@bit.edu.cn

Prof. Changming Wang
superwcm@163.com

# Supplementary Figures and Tables

## Supplementary Figures


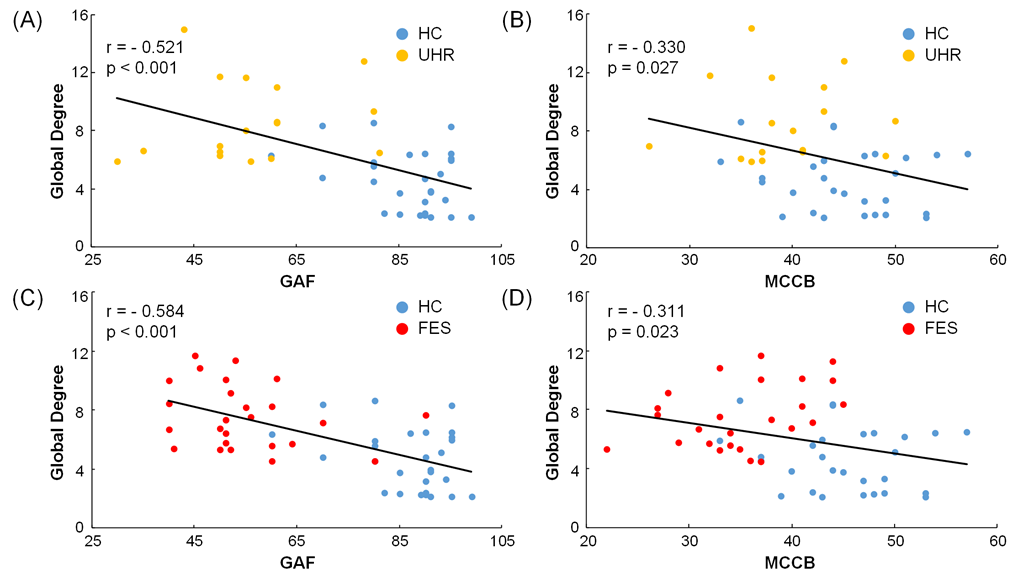


**Supplementary Figure 1.** Correlation of the average global degree and GAF (A and C), MCCB (B and D). The correlations were calculated with two groups: HC and UHR (A and B); HC and FES (C and D). GAF: the Global Assessment of Functioning; MCCB: MATRICS Consensus Cognitive Battery; HC: healthy controls; UHR: ultra-high risk for psychosis; FES: first-episode schizophrenia.

**
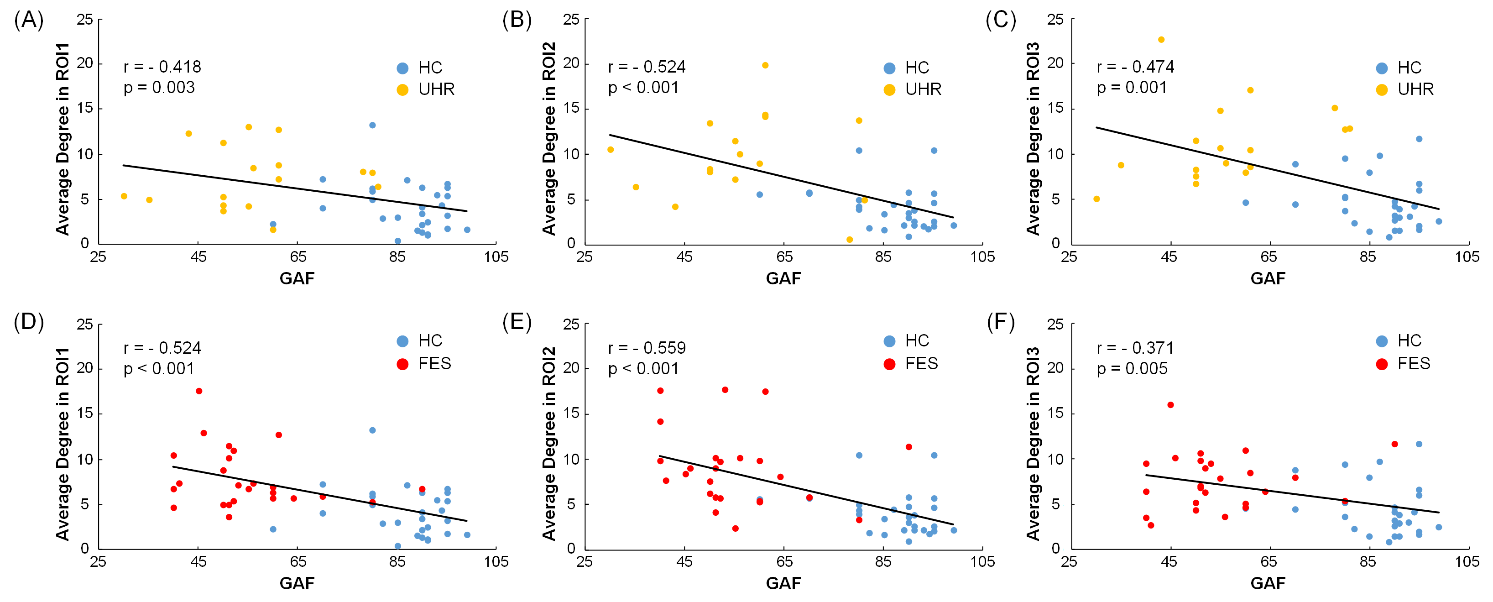
**

**Supplementary Figure 2.** Correlation of the GAF and average degree in ROI1 (A and D), in ROI2 (B and E) and in ROI3 (C and F). The correlations were calculated with two groups: HC and UHR (A, B and C); HC and FES (D, E and F). GAF: the Global Assessment of Functioning; HC: healthy controls; UHR: ultra-high risk for psychosis; FES: first-episode schizophrenia.


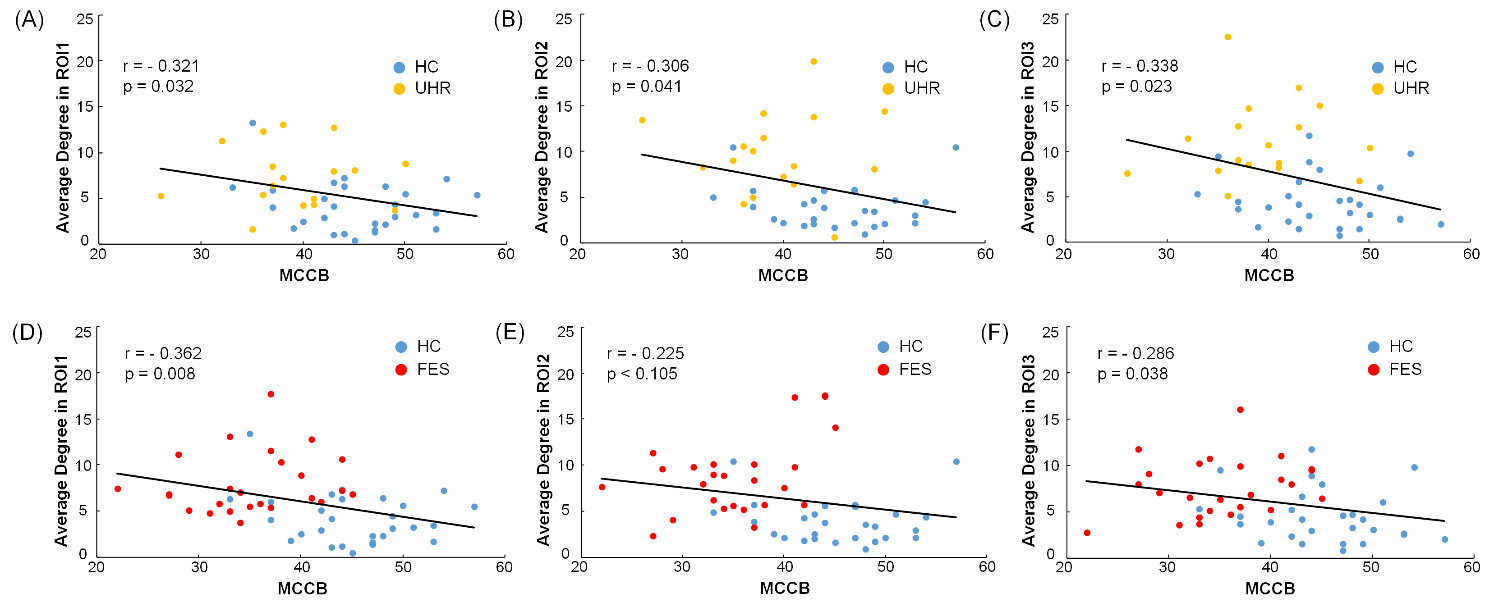


**Supplementary Figure 3.** Correlation of the MCCB and average degree in ROI1 (A and D), in ROI2 (B and E) and in ROI3 (C and F). The correlations were calculated with two groups: HC and UHR (A, B and C); HC and FES (D, E and F). MCCB: MATRICS Consensus Cognitive Battery; HC: healthy controls; UHR: ultra-high risk for psychosis; FES: first-episode schizophrenia.

## Supplementary Tables

|  |  | GAF | | MCCB | |
| --- | --- | --- | --- | --- | --- |
|  |  | r | p | r | p |
| Global | HC | -0.321 | 0.096 | -0.142 | 0.472 |
|  | UHR | 0.086 | 0.711 | 0.041 | 0.875 |
|  | FES | -0.243 | 0.212 | 0.367 | 0.071 |
| ROI1 | HC | -0.189 | 0.334 | -0.245 | 0.209 |
|  | UHR | 0.105 | 0.652 | 0.005 | 0.984 |
|  | FES | -0.225 | 0.250 | 0.180 | 0.388 |
| ROI2 | HC | -0.295 | 0.127 | -0.081 | 0.681 |
|  | UHR | -0.003 | 0.991 | 0.014 | 0.956 |
|  | FES | -0.201 | 0.305 | 0.485 | 0.014^*^ |
| ROI3 | HC | -0.207 | 0.291 | -0.168 | 0.392 |
|  | UHR | 0.153 | 0.507 | 0.075 | 0.775 |
|  | FES | 0.105 | 0.596 | 0.229 | 0.271 |

**Supplementary Table 1.** Correlation of the degree and GAF, MCCB within one group. Only one significant correlation was found between average degree in ROI2 and MCCB (p < 0.05). GAF: the Global Assessment of Functioning; MCCB: MATRICS Consensus Cognitive Battery; HC: healthy controls; UHR: ultra-high risk for psychosis; FES: first-episode schizophrenia.
